# DCAF13 inhibits the p53 signaling pathway by promoting p53 ubiquitination modification in lung adenocarcinoma

**DOI:** 10.1186/s13046-023-02936-2

**Published:** 2024-01-02

**Authors:** Shan Wei, Jing Xing, Jia Chen, Liping Chen, Jiapei Lv, Xiaofei Chen, Tang Li, Tao Yu, Huaying Wang, Kai Wang, Wanjun Yu

**Affiliations:** 1 Department of Respiratory and Critical Care Medicine, The Affiliated People’s Hospital of Ningbo University (Ningbo Yinzhou People’s Hospital), 251, Baizhang Road, Ningbo, Zhejiang 315040 People’s Republic of China; 2 Department of Respiratory and Critical Care Medicine, The Fourth Affiliated Hospital, School of Medicine, Zhejiang University, Yiwu, Zhejiang 322000 People’s Republic of China

**Keywords:** DDB1 and CUL4 associated factor 13, Lung adenocarcinoma, p53 signaling pathway, Ubiquitination, Protein degradation

## Abstract

**Background:**

Lung cancer is a malignant tumor with the highest mortality worldwide. Abnormalities in the ubiquitin proteasome system are considered to be contributed to lung cancer progression with deleterious effects. DDB1 and CUL4 associated factor 13 (DCAF13) is a substrate receptor of the E3 ubiquitin ligase CRL4, but its role in lung cancer remains unknown. In this study, we aimed to investigate the regulatory mechanisms of DCAF13 in lung adenocarcinoma (LUAD).

**Methods:**

So as to investigate the effect of DCAF13 on lung adenocarcinoma cell function using in vivo and in vitro. Mechanistically, we have identified the downstream targets of DCAF13 by using RNA-sequencing, as well as ubiquitination assays, co-immunoprecipitation, immunofluorescence, immunohistochemistry and chromatin immunoprecipitation - qPCR experiments.

**Results:**

Our findings reveal that DCAF13 is a carcinogenic factor in LUAD, as it is highly expressed and negatively correlated with clinical outcomes in LUAD patients. Through RNA-sequencing, it has been shown that DCAF13 negatively regulates the p53 signaling pathway and inhibits p53 downstream targets including p21, BAX, FAS, and PIDD1. We also demonstrate that DCAF13 can bind to p53 protein, leading to K48-linked ubiquitination and degradation of p53. Functionally, we have shown that DCAF13 knockdown inhibits cell proliferation and migration. Our results highlight the significant role of DCAF13 in promoting LUAD progression by inhibiting p53 protein stabilization and the p53 signaling pathway. Furthermore, our findings suggest that high DCAF13 expression is a poor prognostic indicator in LUAD, and DCAF13 may be a potential therapeutic target for treating with this aggressive cancer.

**Conclusions:**

The DCAF13 as a novel negative regulator of p53 to promote LUAD progression via facilitating p53 ubiquitination and degradation, suggesting that DCAF13 might be a novel biomarker and therapeutical target for LUAD.

**Supplementary Information:**

The online version contains supplementary material available at 10.1186/s13046-023-02936-2.

## Background

Lung cancer is the leading cause of cancer death and the second most commonly diagnosed cancer behind female breast cancer in 2020 global cancer statistics, with an estimated 2.2 million new cancer cases and 1.8 million deaths [1, 2]. Non-small cell lung cancer (NSCLC) is the predominant histological type, representing 80–85% of lung tumors. Lung adenocarcinoma (LUAD) is the most predominant subtype of NSCLC, accounting for approximately half of cases [3, 4]. Most patients with lung cancer are diagnosed at the incurable, advanced stage and contribute to the majority of lung cancer deaths. Despite numerous advances in treatments over the past decade, including improved local therapy with surgery or radiation, the use of adjuvant chemotherapy, molecular targeted therapy and immunotherapy for patients with NSCLC, LUAD remains an incurable disease for most patients [5–8]. Thus, a better understanding of responsible mechanism is critical and urgent to identify a therapeutic target for LUAD treatment.

Protein degradation and stabilization are essential for the development of lung adenocarcinoma, and the ubiquitin-proteasome system is the most important protein degradation modality [9, 10]. Protein degraders designed based on the ubiquitin-proteasome system provide tremendous advantages in the orientation of small molecule drugs, especially in cancer therapy [11, 12]. Cullin 4-RING E3 ubiquitin ligase (CRL4) has been proven to be a promise in treating hematological malignancies [13], but its specific substrate molecular and mechanisms still need to be further explored.

DDB1 and CUL4 associated factor 13 (DCAF13) is the substrate recognition receptor belonging to the E3 ubiquitin ligase CRL4 complex. Also, CRL4 comprises the scaffolding proteins cullin 4 A (CUL4A) or cullin 4B (CUL4B), the zinc finger protein RING-box protein 1 (RBX1) and the articulation protein damage specific DNA binding protein 1 (DDB1) [14, 15]. DCAF13 has been identified to participate in various biological processes by specifically modulating the ubiquitination of target substrates, including early embryonic development [16], oocyte meiotic resumption and growth [17, 18], endometrial decidualization [19], immune regulation [20], and tumor proliferation or metastasis [21–25]. For example, DCAF13-CRL4B promotes osteosarcoma cell growth by mediating the ubiquitination of PTEN [21, 22]. Additionally, DCAF13 promotes metastasis in triple-negative breast cancer by mediating the degradation of *DTX3* mRNA [23]. Nevertheless, the functions and mechanisms of DCAF13 are not yet disclosed in LUAD.

Tumor protein p53 (p53) is the most important tumor suppressor and involved in cell signaling in a variety of processes [26], including cell cycle arrest, DNA repair, apoptosis, senescence, autophagy, immunity, ferroptosis, or metabolism [27, 28]. The most important role of p53 as a transcription factor is considered to directly regulate the expression of ~ 500 target genes [29–31], such as *CDKN1A* (encoding cyclin-dependent kinase inhibitor 1, also known as p21 protein) [32], *BBC3* (encoding Bcl-2-binding component 3) [33], *BAX* (encoding BCL2 associated X, apoptosis regulator) [34], *FAS* [35] and some other target genes so as to suppress tumor development. As a nuclear transcription factor, p53 does not possess typical drug target features and has therefore long been considered to be undruggable [36]. Therefore, it will be interesting to investigate its negative regulators, which could be even used as alternative gene therapy strategies.

In this study, we identified that DCAF13 was upregulated and associated with poor prognosis in LUAD patients. Intriguingly, we identified DCAF13 as a novel negative regulator of p53 to promote LUAD progression via facilitating p53 ubiquitination and degradation by cooperatively with the CRL4 complex. We also addressed that DCAF13 modified histone modifications and transcription of p53 downstream target genes. Our studies shed new insights into the mechanism and function of DCAF13 in LUAD development.

## Methods

### Public database analysis

The mRNA expression levels of *DCAF13* in both Cancer Genome Atlas (TCGA) and Genotype-Tissue Expression (GTEx) samples were analyzed using the Gene Expression Profiling Interactive Analysis 2 (GEPIA2) and The University of Alabama at Birmingham CANcer data analysis Portal (UALCAN) databases. Furthermore, the *DCAF13* promoter methylation levels in TCGA samples and protein expression in Clinical Proteomic Tumor Analysis Consortium (CPTAC) samples were analyzed using the UALCAN databases. The correlation between *DCAF13* expression and p53 target genes was evaluated using the GEPIA2 database. The relationship between *DCAF13* expression and prognosis was assessed using the GEPIA2 and KM-plotter databases.

### Tissue microarray, immunohistochemistry (IHC) and clinical samples of LUAD

Tissue microarray was purchased from SHANGHAI OUTDO BIOTECH Company (No. HLugA180Su08), containing 180 samples, which includes 75 pairs of LUAD samples and matched normal samples, 15 LUAD samples without matched adjacent non-cancerous samples, and others). To detect DCAF13 expression in the tissue microarray, we used the High Sensitive and Rapid Immunohistochemical Kit (pH 9.0) (ImmunoWay Biotechnology Company #RS0033) for IHC experiments. All procedures were performed following the instructions of the kit. The antibodies used are listed in Supplementary Table S1.

The clinical samples were collected from The Affiliated People’s Hospital of Ningbo University. Our study was approved by the hospital’s medical ethics committee. Informed consent was obtained from patients for the collection of all clinical samples.

### Western blot

The cells were harvested with a cell scraper and cellular proteins were extracted using RIPA lysis buffer (Beyotime #P0013K) supplemented with a protease inhibitor cocktail (Sangon Biotech, #C500017) at 4℃ for 30 min. The lysates were centrifuged at 14,000 g for 15 min at 4℃ and the resulting supernatant was collected. The total protein was quantified using the BCA protein assay kit (Beyotime #P0012). Protein expression was determined using western blotting. Briefly, after SDS-PAGE using the PAGE Gel Fast Preparation Kit (Epizyme, #0359D300, Shanghai, China), the proteins were transferred to polyvinylidene difluoride (PVDF) membranes using NcmBlot Rapid Transfer Buffer (NCM Biotech, #WB4600, Suzhou, China). The membranes were blocked with Rapid Blocking Solution (NCM Biotech, #P30500, Suzhou, China) for 10 min, and then incubated overnight with antibody dilutions (NCM Biotech, #WB100D, Suzhou, China) mixed with specific primary antibodies. The membrane was washed 3 times with TBST for 10 min each time. Secondary antibodies were incubated at room temperature for one hour, and the immunoblot bands were visualized using the Clarity™ Western ECL Substrate (BIO-RAD #170–5061). The antibodies used in this study are listed in Supplementary Table S1.

### Cell culture and transfection

The human lung cancer cell lines A549, SPC-A1, and NCI-H1299 were obtained from the Chinese Academy of Sciences (Shanghai) Cell Bank and cultured in Dulbecco’s Modified Eagle Medium or RPMI-1640 medium supplemented with 10% fetal bovine serum (FBS) and 1% penicillin/streptomycin. All cells were maintained in a 37 °C humidified incubator with 5% CO_2_.

Small interfering RNA was purchased from GenePharma (Shanghai, China). The sequences of the siRNAs are listed in Supplementary Table S1. The FLAG-DCAF13 (mouse) plasmid was kindly provided by Prof. Hengyu Fan. The transfection procedure of siRNAs or plasmids followed the instructions of the GP-transfect-Mate transfection reagent (GenePharma, Shanghai, China) or Lipo8000™ transfection reagent (Beyotime Biotechnology, Shanghai, China). Plasmid extraction was carried out using a plasmid extraction kit (Vazyme, # DC203-01, Nanjing, China).

The knockdown lentivirus of DCAF13 was generated by Genechem (Shanghai, China) with the sequence of siDCAF13#1. The lentivirus was transfected according to the instructions in A549 cells. We screened shDCAF13 stable knockdown and shControl A549 cells using puromycin.

### Reverse transcription and real-time fluorescence quantification PCR (qPCR)

Cells were collected 24 h after transfection for RNA extraction. Total RNA was extracted from cells using the TransZol Up Plus RNA Kit (TransGen Biotech #ER501-01, Beijing, China) and cDNA was synthesized using the HiScript III All-in-one RT SuperMix Perfect for qPCR (Vazyme, #R333-01, Nanjing, China) according to the manufacturer’s instructions. RT-qPCR was performed on an Applied Biosystems® 7500 Fast instrument (Applied Biosystems, USA) in an Eco 96-well plate, using Taq Pro Universal SYBR qPCR Master Mix (Vazyme #Q712, Nanjing, China). Each PCR reaction consisted of a volume of 20 μl, containing 10 μl of 2×Master mix, 0.4 μl of Primer1 (10μM), 0.4 μl of Primer2 (10μM), and x μl of template cDNA and ddH2O, which were loaded into one well. The primer sequences used are listed in Supplementary Table S1.

### Cell colony formation experiments

To investigate the impact of DCAF13 on colony formation, we employed three different siRNAs to knock down DCAF13 expression. 48 h after transfection, we seeded 1000 cells in each 3.5-cm dish. After 14 days of culture, we fixed the cells with paraformaldehyde for 15 min, stained them with 0.1% crystal violet for 15 min, and captured images.

### CCK-8 experiments

A CCK-8 assay kit (TransGen Biotech # FC101-03, Beijing, China) was used to determine the effect of DCAF13 on cell proliferative abilities. Three siRNAs and siControl were transfected to knock down the expression of DCAF13 according to the manufacturer’s protocol. Cells were used for plate spreading 48 h after transfection. 2 × 10^^3^ transfected cells with 100 μl of culture medium were seeded and grown in 96-well plates, followed by incubation with CCK-8 solutions for 1 h at indicated periods (0, 2, 4, and 6 days). Subsequently, the absorbance value (450 nm) was measured using a microplate reader.

### Apoptosis experiments

The Annexin V-FITC/PI Apoptosis Detection Kit (Vazyme #A211, Nanjing, China) was used to detect cell apoptosis. Cells were transfected as described above. After trypsin digestion, 1–5 × 10^^5^ cells were collected and centrifuged at 1000 rpm, 4 °C for 5 min, and the supernatant was discarded. The cells were washed twice with pre-cooled PBS and resuspended in 100 μl of 1 × Binding Buffer. Annexin V-FITC (5 μl) and PI Staining Solution (5 μl) were added to the cells for staining (protected from light and incubated at room temperature (20 ~ 25 °C) for 10 min). Then, 400 μl of 1 × Binding Buffer was added and gently mixed. The stained cells were detected by flow cytometry within 1 h.

### Transwell experiments

To investigate the effect of DCAF13 on cell migration ability, we transfected cells with siRNA as described above. After 48 h, we seeded 2 × 10^^4^ cells in the upper chamber of a transwell and suspended them in 100 μl medium supplemented with either serum-free or 10% serum. The bottom chamber was added with 600 μl medium supplemented with 20% serum. After 24 h, we fixed the cells in the chamber with 95% absolute ethanol for 15 min, stained them with 0.1% crystal violet for 15 min, and photographed them.

### High-throughput mRNA sequencing (mRNA-seq)

The mRNA sequencing technology was supported by Majorbio (Shanghai, China). All raw data have been uploaded to Sequence Read Archive (SRA), BioProject: PRJNA947744. In brief, total RNA was extracted from the tissue using TRIzol® Reagent according to the manufacturer’s instructions (Invitrogen), and genomic DNA was removed using DNase I (TaKara). Only high-quality RNA samples (OD260/280 = 1.8 ~ 2.2, OD260/230 ≥ 2.0, RIN ≥ 8.0, 28 S:18 S ≥ 1.0, > 1 μg) were used to construct the sequencing library. RNA purification, reverse transcription, library construction, and sequencing were performed at Shanghai Majorbio Bio-pharm Biotechnology Co., Ltd. (Shanghai, China) following the manufacturer’s instructions (Illumina, San Diego, CA). The transcriptome library was prepared using the TruSeq TM RNA sample preparation Kit from Illumina (San Diego, CA) using 1 μg of total RNA. After quantification by TBS380, paired-end RNA-seq sequencing libraries were sequenced with the Illumina NovaSeq 6000 sequencer (2 × 150 bp read length).

The data were analyzed on the online platform of Majorbio Cloud Platform. To identify DEGs (differential expression genes) between two different groups, the expression level of each gene was calculated using the transcripts per million reads (TPM) method. RSEM (http://deweylab.biostat.wisc.edu/rsem/) [37] was used to quantify gene abundances. Differential expression analysis was performed using DESeq2 /DEGseq /edgeR /Limma /NOIseq [38–41]. In addition, functional enrichment analysis, including GO (Gene Ontology, http://www.geneontology.org) and KEGG [42] (Kyoto Encyclopedia of Genes and Genomes, http://www.genome.jp/kegg/) were performed to identify which DEGs were significantly enriched in GO terms and metabolic pathways at P-adjust ≤ 0.05 compared with the whole-transcriptome background. GO functional enrichment and KEGG pathway analysis were carried out by Goatools (https://github.com/tanghaibao/Goatools) and KOBAS (http://kobas.cbi.pku.edu.cn/home.do) [43].

### Chromatin immunoprecipitation - qPCR (ChIP - qPCR) assay

ChIP-qPCR was conducted following previously described methods [44]. Briefly, shDCAF13 and shControl A549 cells were fixed with 1% formaldehyde and then quenched with glycine. DNA was fragmented into lengths of 200 ~ 1000 bp using an ultrasonic cell disruptor after adding 1 mL of SDS lysis solution. Protein A/G agarose beads (20 μL) were added to remove non-specific binding proteins. Next, the supernatant was added with 1–4 immunoprecipitating antibodies and shaken gently at 4 °C overnight. An additional 50 μL of supernatant without antibodies was prepared as a negative control. Then, 40 μL of Protein A/G agarose beads were added to form antibody-protein A immune complexes. Immunoprecipitation complexes were washed sequentially with low-salt buffer, high-salt buffer, LiCl buffer, and TE buffer. The beads were eluted three times by adding 200 μL of ChIP elution solution to obtain 600 μL of eluate. The sample was then added with 24 μL of 5 M NaCl and incubated at 65 °C overnight to remove the formaldehyde crosslink. DNA purification was performed by phenol/chloroform extraction. DNA fragments were analyzed using qPCR. Primer sequences are provided in Supplementary Table S1. The data presented represent the mean ± SD from three independent experiments.

### Immunofluorescence staining

Cells were fixed with 4% formaldehyde and then permeabilized with Triton X-100 (Solarbio #P9600, Beijing, China) for 15 min, followed by three washes with PBS. After blocking with 1% serum (NEOBIOSCIENCE #NB-AG-10, Shenzhen, China) for 1 h, cells were washed twice with PBS and then incubated with p53 and DCAF13 antibodies overnight at 4 °C, followed by incubation with fluorescent secondary antibody for 1 h the next day. Finally, the cells were mounted with VECTASHIELD Antifade Mounting Medium with DAPI (Vector Laboratories, Burlingame, CA, USA). The antibodies used are listed in Supplementary Table S1.

### Co-immunoprecipitation (Co-IP) experiments

For Co-IP experiments, whole cell lysates were extracted and an equal amount of protein was pre-cleared with Protein A/G PLUS-Agarose for 1 h (Santa Cruz, sc-2003). Supernatants were then incubated with anti-p53, anti-DCAF13, Rabbit Control IgG (AC005), or Mouse Control IgG (AC011) for 4 h, and the immunoprecipitated proteins were collected with Protein A/G PLUS-Agarose overnight at 4 °C with rotation. Immune complexes were subsequently washed three times and analyzed using western blot with indicated antibodies. To avoid the noise of heavy chains, the secondary antibodies used for the Co-IP assays were IPKine™ HRP, Mouse Anti-Rabbit IgG LCS (Abbkine, #A25022) and IPKine™ HRP, Goat Anti-Mouse IgG LCS (Abbkine, A25012).

### Ubiquitination experiments

A549 and SPC-A1 cells transfected with HIS-ubiquitination plasmid with or without DCAF13 knockdown were lysed using RIPA lysis buffer. After reserving a portion of the cell lysate for input analysis, the remaining cell lysate was pre-cleared with Protein A/G PLUS-Agarose (Santa Cruz, sc-2003) at 4 °C for 1 h. The supernatants were then incubated with p53 antibody for 4 h on a rotating device at 4 °C, and the immunoprecipitated proteins were collected with Protein A/G PLUS-Agarose overnight on a rotating device at 4 °C. The next day, the immunoprecipitated proteins were washed with PBS 3 times and boiled for 10 min in 2X loading buffer. Proteins were resolved by western blotting and immunoblotted with the indicated antibodies.

### Tumor growth assays in vivo

All animal experiments were conducted in compliance with the ethical standards set forth by the Animal Nursing Committee of the Medical College of Ningbo University. The DCAF13 knockdown virus was purchased from Genechem (Shanghai, China) with the sequence siDCAF13#1 and transfected into A549 cells according to the instructions. Stable knockdown of DCAF13 was achieved using puromycin in the shDCAF13 group, while shControl was used as the control group, then 2 × 10^^6^ cells suspended in 100 μl of 1:1 PBS and Matrigel (Corning, NY, USA) were subcutaneously injected into BALB/c mice (Beijing Vital River Laboratory Animal Technology Company, Beijing, China). Tumor volume was measured weekly using the formula (length × width ^2^)/2. Four weeks later, the mice were humanely euthanized, and the tumors were collected for further analysis.

### Statistical analysis

All data were analyzed using the statistical software GraphPad Prism 8.0 (GRAPH PAD SOFTWARE Inc., CA, USA). The data are presented as mean ± standard deviation (SD). Comparisons between groups were determined by the student’s t-test, the Mann–Whitney U test, or log-rank test (for Kaplan-Meier analysis), and multiple group comparisons were analyzed by one-way ANOVA. The relationship between DCAF13 protein levels and clinicopathological parameters was determined using the chi-square test. Spearman correlation analysis was used to calculate the correlation between DCAF13 and other gene expression. Statistical significance was set at *p < 0.05, ** *p* < 0.01, and *** *p* < 0.001, were considered statistically significant.

## Results

### DCAF13 is highly expressed and a poor prognostic indicator in LUAD

**Fig. 1:**
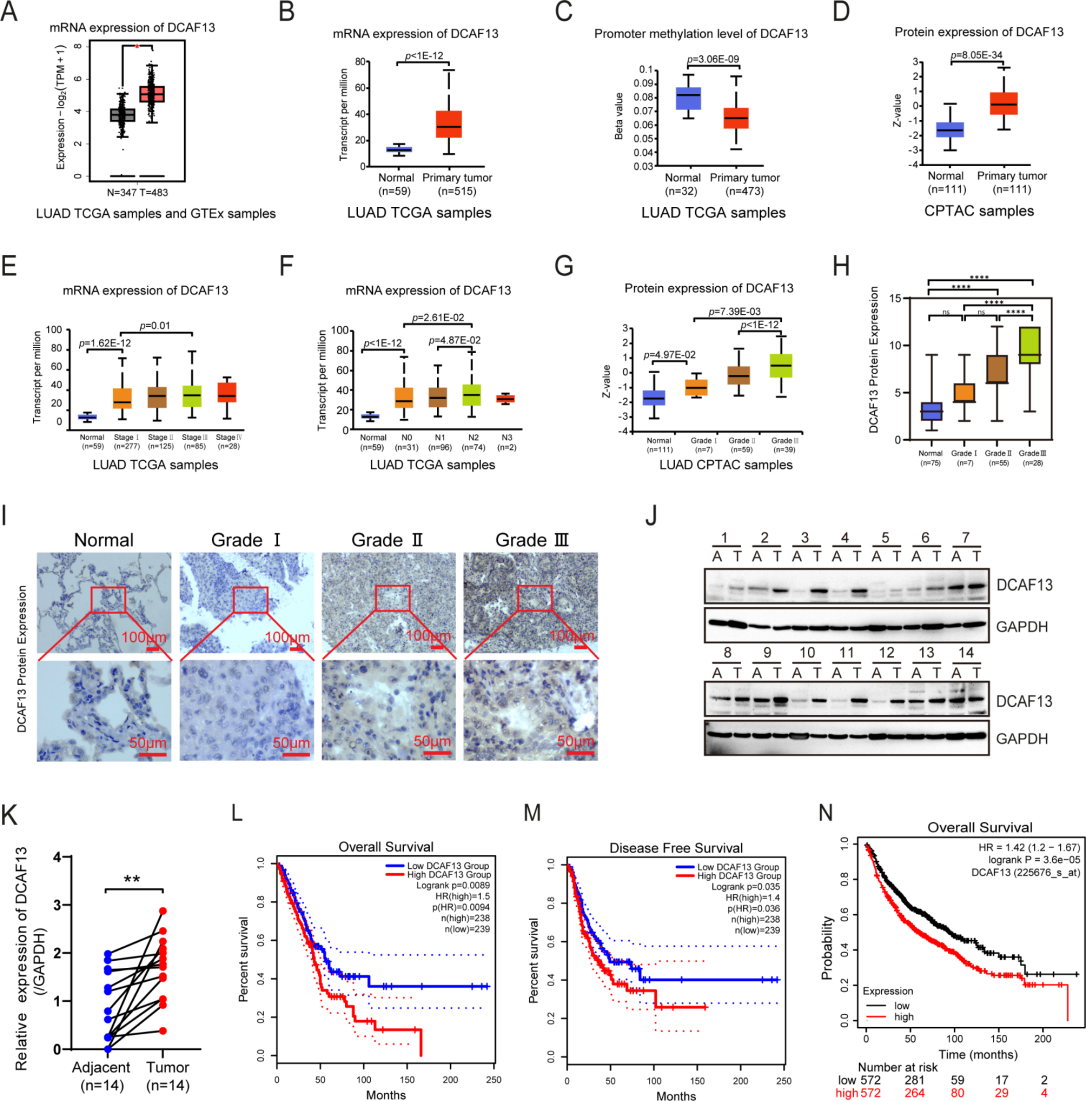
DCAF13 is highly expressed and associated with poor prognosis in LUAD. **A**. *DCAF13* mRNA expression by GEPIA2. **B-G**. DCAF13 mRNA expression, promoter methylation level, and protein level by UALCAN. Differences in significance were marked (Mann-Whitney U test). **H** and **I**. DCAF13 protein expression levels were detected in a tissue microarray by IHC (Mann-Whitney U test). The scale bar is marked as shown. H, quantification analysis of DCAF13 expression in grade. I, representative DCAF13 immunohistochemical staining. **J** and **K**. DCAF13 is highly expressed in fresh clinical LUAD tissue specimens by Western blotting (K, paired *t*-test). A, Adjacent paraneoplastic tissue; T, Tumor tissue. **L-M**. Association between DCAF13 mRNA expression and prognosis in TCGA LUAD samples by Kaplan-Meier analysis (log-rank test). L (OS), M (DFS). **N**. Association between *DCAF13* mRNA expression and prognosis in lung cancer by KM-plotter database (log-rank test). Data are presented as mean ± SD. * *p* < 0.05, ** *p* < 0.01, *** *p* < 0.001, and **** *p* < 0.0001

DCAF13 is a substrate recognition protein in CRL4 E3 ligase and its role is not yet clear in LUAD progression. We performed an analysis of DCAF13 expression and its correlation with prognosis in TCGA LUAD samples using the GEPIA2 or UALCAN database. The results suggested that *DCAF13* mRNA was significantly elevated in LUAD tissues compared with normal lung tissues (*p* < 0.05, Fig. 1A and *p* < 1e-12, 1B), whether *TP53* is mutated or not (Supplementary Fig. S1A). Additionally, *DCAF13* promoter methylation level was significantly lower in LUAD (*p* = 3.06e-09, Fig. 1C), whether *TP53* is mutated or not (Supplementary Fig. S1B). Meanwhile DCAF13 protein levels were significantly elevated in CPTAC LUAD samples (*p* = 8.05e-34, Fig. 1D). Moreover, *DCAF13* mRNA expression was positively correlated with clinical stage or N stage (Fig. 1E-F) and protein levels were positively correlated with grade (Fig. 1G). We wondered whether DCAF13 protein levels were consistent with the results from the public databases. Thus, we validated the results in a tissue microarray with 165 LUAD specimens (Fig. 1H and I). 70% tumors of 90 LUAD specimens expressed high levels of DCAF13 protein; in contrast, high DCAF13 expression was found in only 17.3% of 75 noncancerous tissues (*p* < 0.001, Table 1), we evaluated whether DCAF13 expression was associated with clinicopathological parameters in LUAD patients. The results showed that high DCAF13 expression was positive correlation with grade, irrelevant to gender, age, stage, etc. (Fig. 1H-I, and *p* > 0.05, Table 2). We similarly demonstrate that DCAF13 protein is highly expressed in 14 pairs of matched fresh clinical LUAD samples (*p* < 0.01, Fig. 1J-K).

**Table 1:** DCAF13 expression in LUAD and noncancerous tissues

Group	Case	DCAF13 expression		*P*-value^a^
	n = 165	**Low**	High	
LUAD	90	27 (30%)	63 (70%)	**<0.001**
noncancerous tissues	75	62 (82.7%)	13 (17.3%)	

^a^ Chi-square test

**Table 2:** Relationship between DCAF13 expression and clinicopathological characteristics

**Group**		Case(percent)		**DCAF13 expression**		*p* value^a^
				low	high	
gender n = 90	total	90		27	63	
	female	36	(40.00%)	14	22	0.162
	male	54	(60.00%)	13	41	
age n = 90	total	90		27	63	
	≤ 55	23	(25.56%)	4	19	0.187
	>55	67	(74.44%)	23	44	
grade n = 90	total	90		27	63	
	1	7	(7.78%)	5	2	**0.010**
	2	55	(61.11%)	18	37	
	3	28	(31.11%)	4	24	
stage n = 89	total	89		27	62	
	1	31	(34.83%)	11	20	0.797
	2	20	(22.47%)	6	14	
	3	37	(41.57%)	10	27	
	4	1	(1.12%)	0	1	
T n = 86	total	86		26	60	
	1	18	(20.93%)	5	13	0.894
	2	48	(55.81%)	16	32	
	3	15	(17.44%)	4	11	
	4	5	(5.81%)	1	4	
N n = 77	total	77		22	55	
	0	43	(55.84%)	13	30	0.487
	1	14	(18.18%)	5	9	
	2	15	(19.48%)	2	13	
	3	5	(6.49%)	2	3	
EGFR n = 69	total	69		21	48	
	negative	56	(81.16%)	19	37	0.317
	positive	13	(18.84%)	2	11	
ALK n = 73	total	73		22	51	
	negative	59	(80.82%)	20	39	0.204
	positive	14	(19.18%)	2	12	

^a^ Chi-square test

Furthermore, we then investigated the prognostic value of *DCAF13* in LUAD, patients were grouped into high expression group and low expression group basing on *DCAF13* mRNA expression. Kaplan-Meier analysis revealed that patients with higher *DCAF13* expression had shorter survival than those with lower expression regardless of overall survival (OS, *p* = 0.0094) or disease-free survival (DFS, *p* = 0.036) using the GEPIA2 database (Fig. 1L and M). Similarly, Kaplan-Meier analysis from the KM-plotter database revealed that patients with high *DCAF13* expression had a significantly worse survival than those with low *DCAF13* mRNA expression in lung cancer (*p* = 3.6e-05, Fig. 1N). In summary, we discovered that DCAF13 is highly expressed in LUAD, and high *DCAF13* expression group was a poor prognostic indicator for LUAD.

### DCAF13 knockdown inhibits the growth and migration and promotes the apoptosis of LUAD cells

**Fig. 2:**
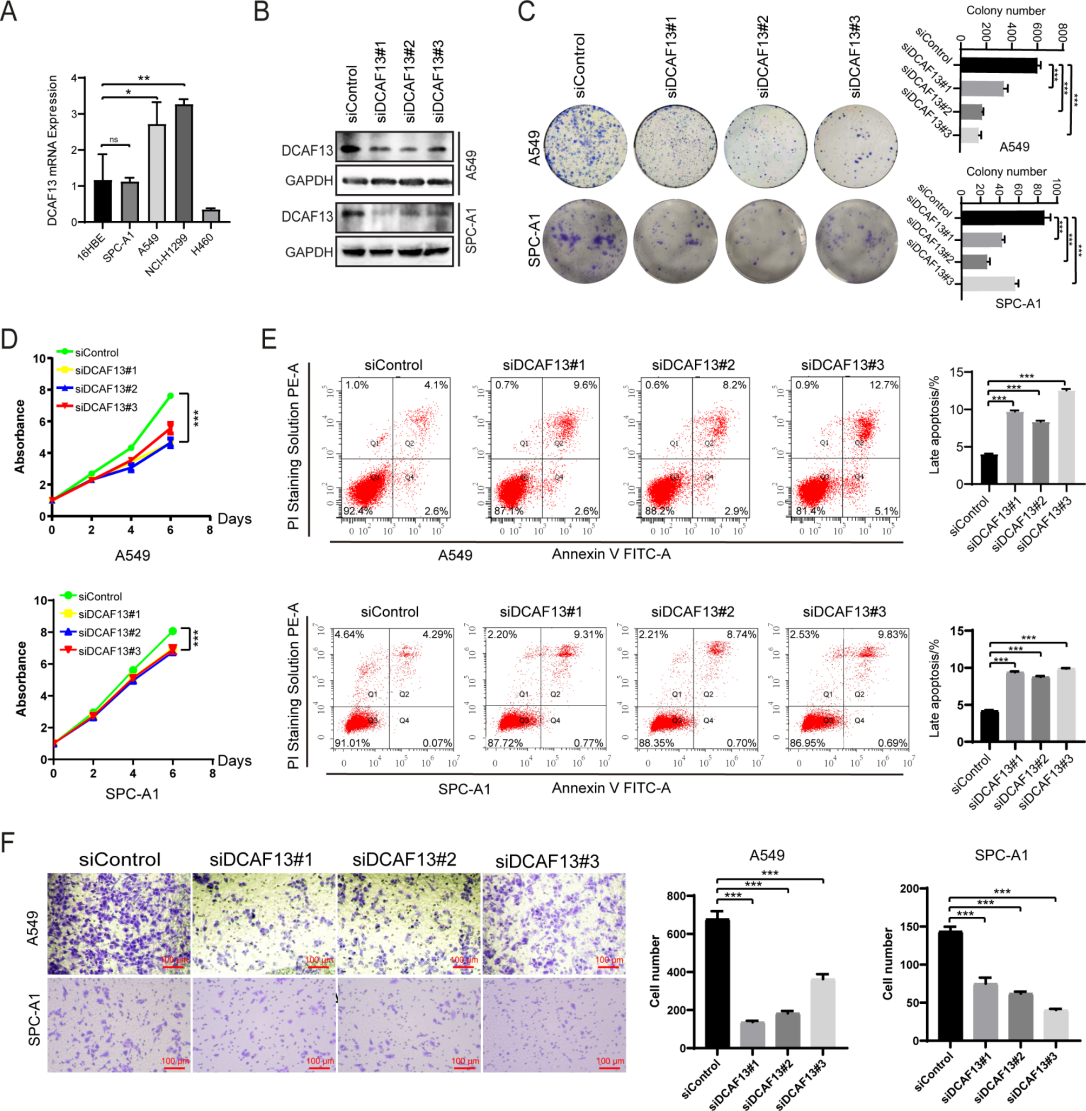
DCAF13 knockdown inhibits LUAD cell growth, migration and apoptosis inhibition in *vitro*. (**A**) *DCAF13* mRNA expression was detected in LUAD cell lines. The 16HBE cells are normal lung bronchial epithelial cells. A549, SPC-A1, NCI-H1299 cells are LUAD cell lines. H460 cells are human large-cell lung cancer cells. (**B**) Effective interference of DCAF13 expression by three small interfering RNAs, siDCAF13#1, #2, #3, in A549 and SPC-A1 cells. (**C**) Cell clone formation experiments were performed in A549 and SPC-A1 cells with DCAF13 knockdown. (**D**) CCK-8 experiments were performed in A549 and SPC-A1 cells. (**E**) Flow cytometry was used to analyze the apoptosis rates in A549 and SPC-A1 cells with DCAF13 knockdown, and the percentage of late apoptosis was compared. (**F**) Cell migration experiments were performed in A549 and SPC-A1 cells with DCAF13 knockdown. Student’s t-test was used in Fig. 2A, C-F. Data presented as mean ± SD, n = 3, * *p* < 0.05, ** *p* < 0.01, and *** *p* < 0.001. Scale bar = 100 μm

To investigate the role of DCAF13 in LUAD progression in vitro, we first detected the endogenous expression of DCAF13 in LUAD cell lines and normal lung bronchial epithelial cells. We found that DCAF13 mRNA expression was significantly overexpressed in LUAD cell lines (Fig. 2A). We used three small interfering RNAs siDCAF13#1, #2, #3 or FLAG-DCAF13 plasmids to effectively knock down or overexpress DCAF13 expression in A549 and SPC-A1 cells (Fig. 2B and Supplementary Fig. S2A). Subsequently, we performed a series of biological functional experiments. The clone formation assay confirmed that DCAF13 knockdown inhibited the cell clone formation capability in A549 and SPC-A1 cells (Fig. 2C), while DCAF13 overexpression significantly promoted cell clone formation ability (Supplementary Fig. S2B). The CCK-8 experiments demonstrated that DCAF13 knockdown significantly inhibited cell growth in A549 and SPC-A1 cells (Fig. 2D), while DCAF13 overexpression significantly promoted cell growth (Supplementary Fig. S2C). The flow cytometry results evidenced that DCAF13 knockdown promoted cell advanced apoptosis in A549 and SPC-A1 cells (Fig. 2E), while DCAF13 overexpression significantly inhibited cell advanced apoptosis (Supplementary Fig. S2D). The transwell experiment showed that DCAF13 knockdown remarkably inhibited the cell migration capability in A549 and SPC-A1 cells (Fig. 2F), while DCAF13 overexpression significantly promoted cell migration capability (Supplementary Fig. S2E). All these findings concluded that downregulation of DCAF13 inhibited the malignant biological behavior of LUAD cells.

### DCAF13 negatively regulates the p53 signaling pathway

**Fig. 3:**
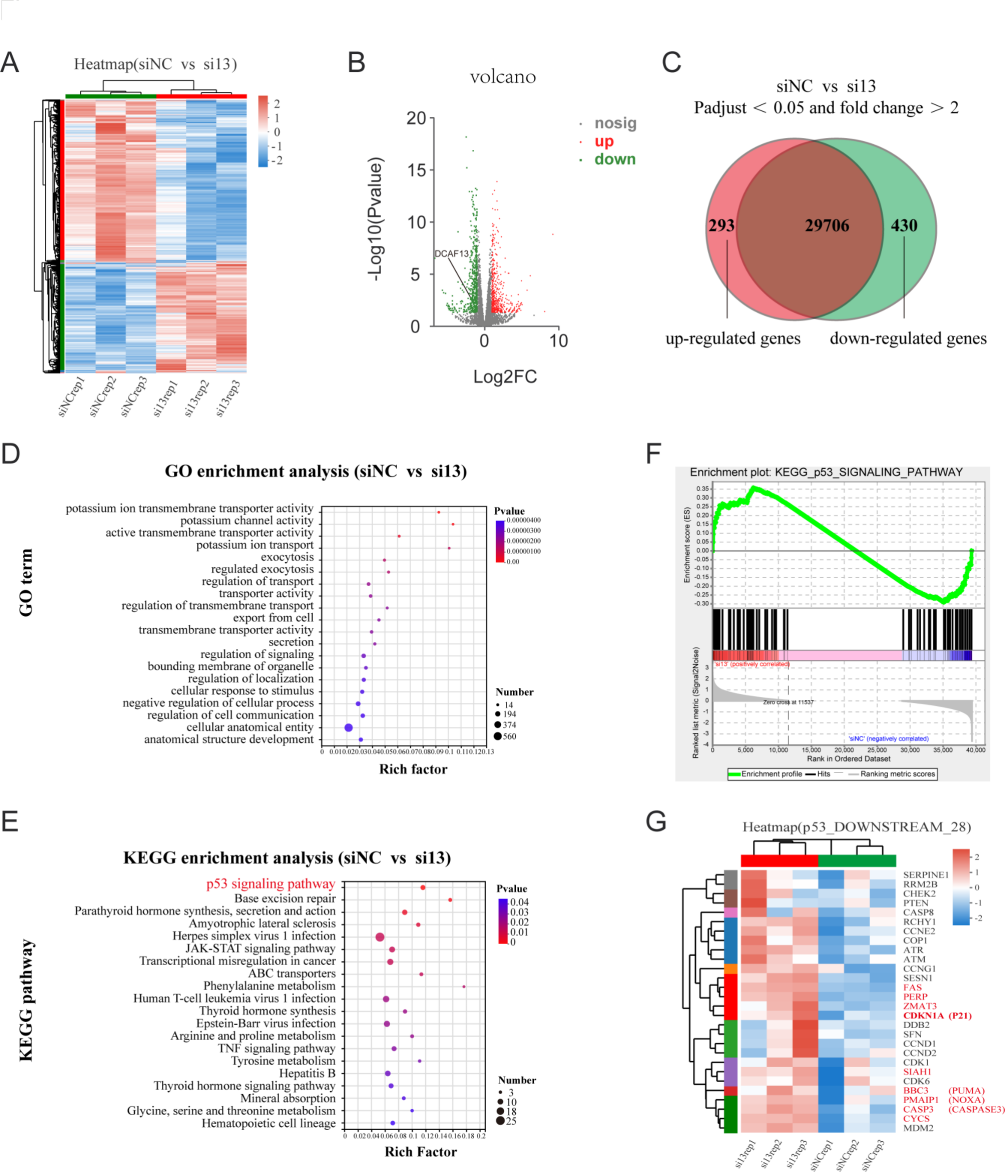
DCAF13 negatively regulates the p53 signaling pathway. **A-C**. Heat map, volcano map and venn diagram of differential gene expression in A549 cells followed by DCAF13 knockdown (*p*-adjust< 0.05 and fold change > 2). **D**. Bubble map of top 20 pathways for all differential genes by GO enrichment analysis. **E**. Bubble map of top 20 pathways for all differential genes by KEGG enrichment analysis. **F**. Gene Set Enrichment Analysis (GSEA) of the p53 signaling pathway. **G**. Heat map of p53 downstream 28 genes acting core in the Fig. 3F

We have tentatively demonstrated that DCAF13 is overexpressed in LUAD and promotes its malignant progression in vitro. To further investigate the underlying mechanisms, we conducted mRNA-seq after knocking down DCAF13 in A549 cells. We identified 293 up-regulated genes and 430 down-regulated genes with *p*-adjust< 0.05 and fold change > 2 (Fig. 3A-C and Supplementary Table S2). GO enrichment analysis with all differential genes revealed that DCAF13 knockdown significantly affected various cellular processes including transmembrane transporter activity, potassium ion transport, regulated exocytosis, regulation of signaling, regulation of localization, negative regulation of cellular process, regulation of localization, regulation of cell communications etc. (Fig. 3D).

Furthermore, KEGG enrichment analysis with all differential genes showed that the p53 signaling pathway was the most significantly enriched pathway in the mRNA-seq (Fig. 3E), and the differential genes were also enriched in various other pathways including base excision repair, parathyroid hormone synthesis and secretion, amyotrophic lateral sclerosis, herpes simplex virus 1 infection, JAK-STAT signaling pathway, transcriptional misregulation in cancer, ABC transporters, phenylalanine metabolism, and human T-cell leukemia virus 1 infection (Fig. 3E). GSEA enrichment analysis indicated that DCAF13 negatively regulates the p53 signaling pathway (Fig. 3F), and we plotted the expression heat map of the 28 genes that contributed most to the enrichment score which included several tumor suppressor genes such as *CDKN1A, FAS, CYCS, PERP, BBC3, PMAIP1, CASP3, SIAH1, ZMAT3*, etc. (Fig. 3G). Therefore, we propose that DCAF13 inhibits p53 downstream gene expression by negatively regulating the p53 signaling pathway, leading to the promotion of LUAD progression.

### DCAF13 knockdown facilitates the p53 protein levels and its downstream target gene expression

**Fig. 4:**
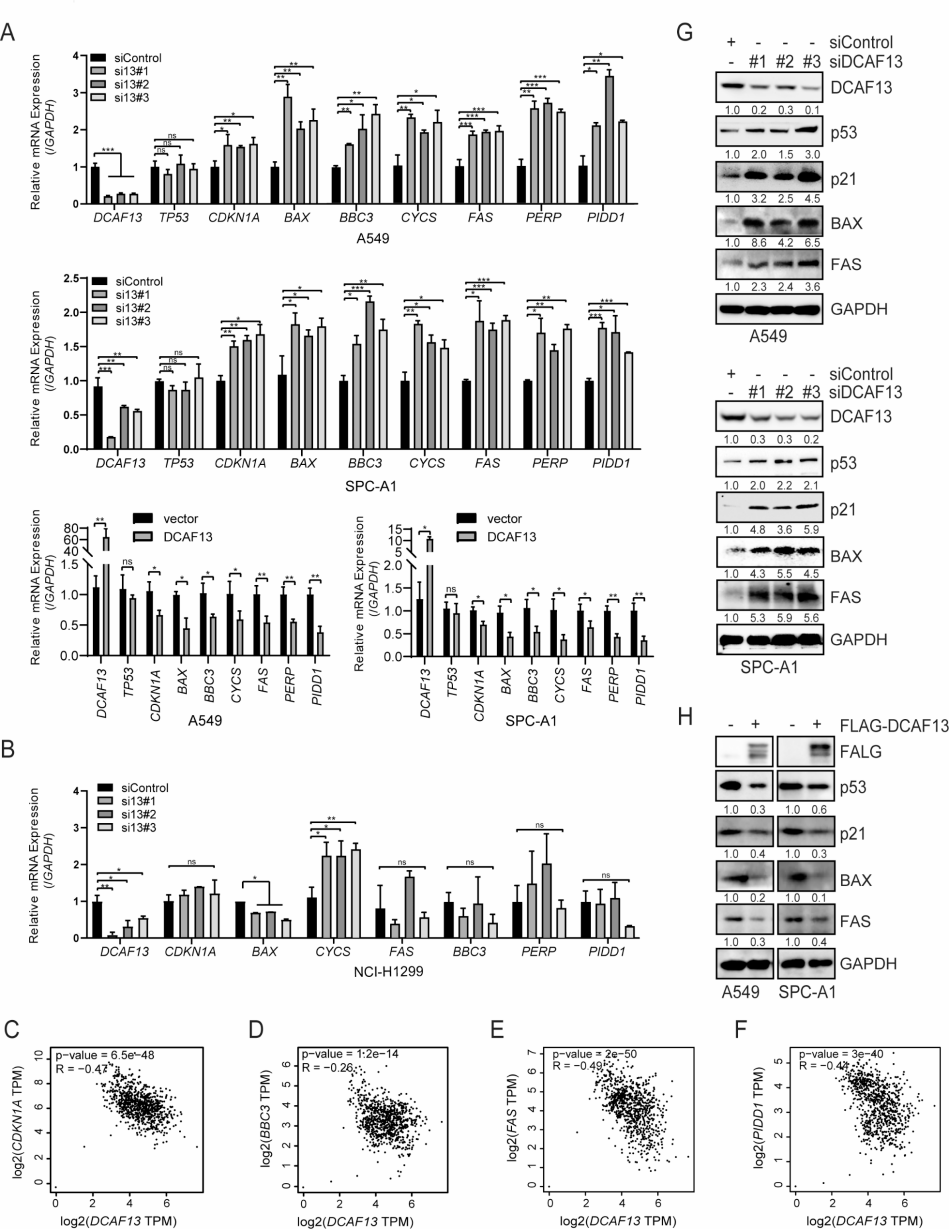
DCAF13 knockdown promotes p53 protein and its downstream target gene expression. (**A**) The mRNA expression of *DCAF13, TP53, CDKN1A, BAX, BBC3, CYCS, FAS, PERP* and *PIDD1* were detected by RT-qPCR in A549 or SPC-A1 cells transfected with the indicated siRNAs or overexpression plasmids (si13 means siRNA for DCAF13). (**B**) The mRNA expression of *DCAF13, CDKN1A, BAX, BBC3, CYCS, FAS, PERP*, and *PIDD1* were detected by RT-qPCR in NCI-H1299 cells transfected with siControl or the indicated siRNAs. *GAPDH* was used as a control. Student’s t-test was used. Data presented as mean ± SD, n = 3, * *p* < 0.05, ** *p* < 0.01, and *** *p* < 0.001. **C-F**. Correlation between *DCAF13* and *CDKN1A, BBC3, FAS, PIDD1* mRNA expression in TCGA LUAD and GTEx samples by GEPIA2 (Spearman correlation coefficient). G-H. Protein expression levels of p53, p21, BAX, FAS were analyzed by Western blotting in A549 or SPC-A1 cells transfected with the indicated siRNAs or overexpression plasmids. GAPDH was used as a control

To investigate whether DCAF13 knockdown negatively regulates the p53 signaling pathway and its downstream genes, we utilized three different small interfering RNAs to knockdown DCAF13 expression in A549, SPC-A1 (p53 wild-type cell lines), and NCI-H1299 (a p53-deficient cell line) cells. RT-qPCR experiments revealed that DCAF13 knockdown did not affect the mRNA expression of *TP53* and *CASP3* but significantly upregulated the mRNA expression of *CDKN1A, BAX, BBC3, CYCS, FAS, PERP*, and *PIDD1* in A549 and SPC-A1 cells (Fig. 4A, Supplementary Fig. S2). Furthermore, we found that DCAF13 overexpression did not affect the mRNA expression of *TP53* but significantly inhibited the mRNA expression of *CDKN1A, BAX, BBC3, CYCS, FAS, PERP*, and *PIDD1* in A549 and SPC-A1 cells (Fig. 4A). However, knockdown of DCAF13 in NCI-H1299 cells only promoted *CYCS* mRNA expression, with no effect on the mRNA expression of *CDKN1A, BBC3, FAS, PERP*, and *PIDD1*, which is inconsistent with the findings in A549 and SPC-A1 cells (Fig. 4B). These conflicting results between different cell types suggest that DCAF13 downregulates *CDKN1A, BAX, BBC3, FAS*, and *PERP* mRNA expression via the p53 signaling pathway. Moreover, we found a significant negative correlation between *DCAF13* and *CDKN1A* (*p* = 6.5e-48, R=-0.47), *BBC3* (*p* = 1.2e-14, R=-0.26), *FAS* (*p* = 2e-50, R=-0.49), and *PIDD1* (*p* = 3e-40, R=-0.44) mRNA expression levels in TCGA-LUAD and GTEx samples using the GEPIA2 database (Fig. 4C-F). Finally, Western blot experiments demonstrated that DCAF13 knockdown increased the protein levels of p53, p21, BAX, and FAS in A549 and SPC-A1 cell lines, while DCAF13 overexpression significantly inhibited the protein levels of p53, p21, BAX, and FAS in A549 and SPC-A1 cell lines (Fig. 4G-H). Therefore, we hypothesize that DCAF13 downregulates the p53 signaling pathway and its downstream target genes by interfering with p53 protein stabilization.

### DCAF13 knockdown alters histone modifications of p53 target genes

**Fig. 5:**
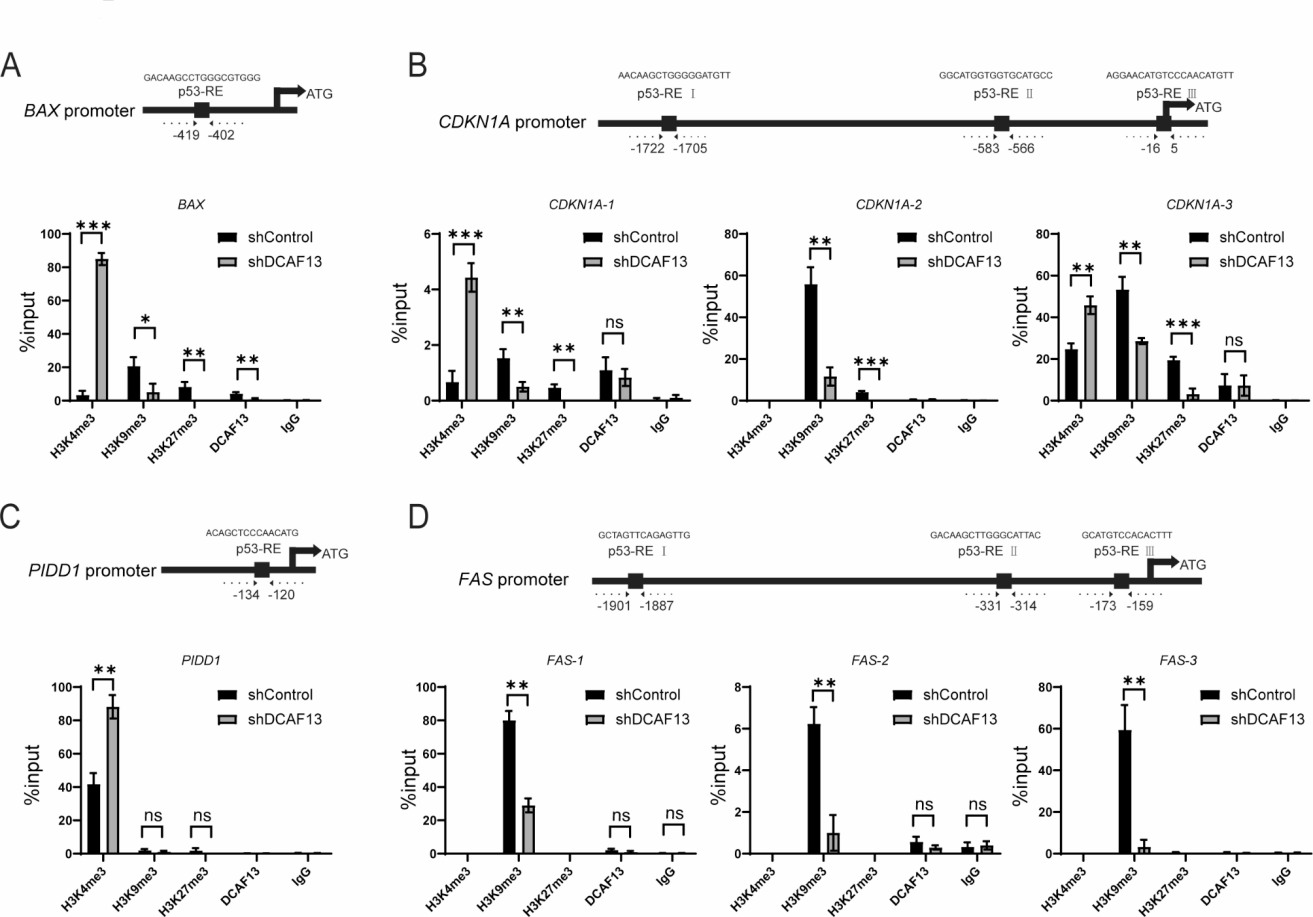
DCAF13 knockdown alters histone modifications of p53 target genes. **A-D**. The p53-RE positions in *BAX, CDKN1A, PIDD1, FAS* promoter regions and alterations of H3K4me3, H3K9me3 or H3K27me3 on p53-RE by ChIP-qPCR in shControl or shDCAF13 A549 cells. Data were statistically analyzed using Student’s t-test and values are shown as mean ± SD. * *p* < 0.05, ** *p* < 0.01, and *** *p* < 0.001

Histone modifications play a crucial role in the regulation of gene transcription, where tri-methylation of histone H3 at lysine 4 (H3K4me3) is associated with gene transcriptional activation, tri-methylation of histone H3 at lysine 9 (H3K9me3) and tri-methylation of histone H3 at lysine 27 (H3K27me3) are linked to gene transcriptional deactivation [45]. To investigate the effect of DCAF13 on histone modifications of p53 target genes, we performed ChIP-qPCR experiments to examine the changes in histone modifications on the promoter region of p53 target genes in A549 cells. We identified one or three p53 response elements (p53-RE) in the promoter regions of *BAX, CDKN1A, PIDD1*, and *FAS* using the JASPAR database (the 9th release of the open-access database of transcription factor binding profiles) [46]. Subsequently, we detected the variations in H3K4me3, H3K9me3, and H3K27me3 on the p53-RE utilizing ChIP-qPCR assays. Our results showed that knockdown of DCAF13 significantly increased H3K4me3 and decreased H3K9me3 or H3K27me3 in the p53-RE regions of the *BAX* and *CDKN1A* promoters (Fig. 5A-B), DCAF13 knockdown remarkably increased H3K4me3 in the p53-RE region of the *PIDD1* promoter (Fig. 5C), and DCAF13 knockdown remarkably decreased H3K9me3 in the p53-RE region of the *FAS* promoter (Fig. 5D). In conclusion, these findings indicate that DCAF13 knockdown promotes p53 target gene expression by upregulating H3K4me3 and downregulating H3K9me3 or H3K27me3.

### DCAF13 specifically recognizes p53 protein as its ubiquitinated substrate

**Fig. 6:**
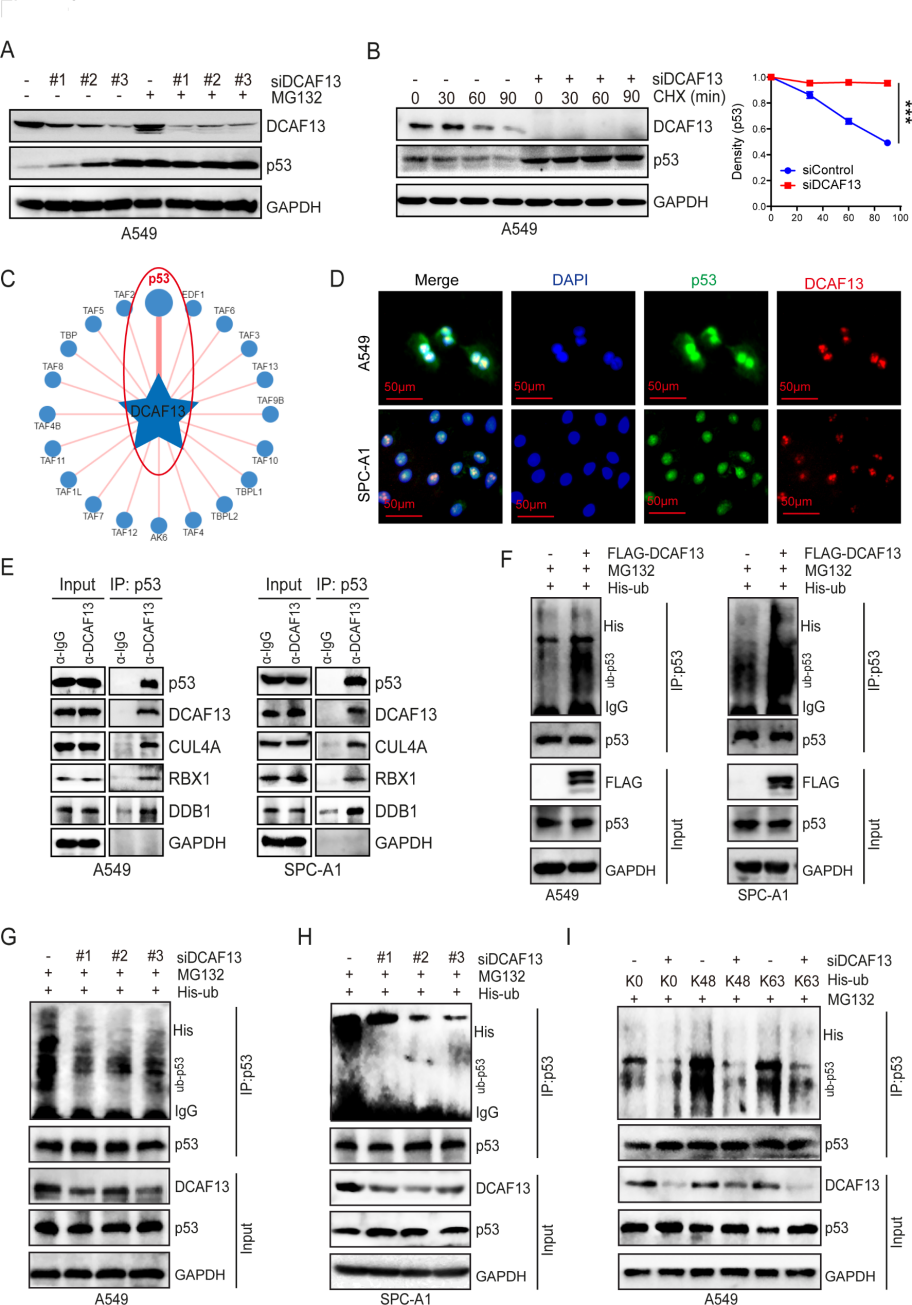
DCAF13 inhibits p53 protein stability via ubiquitination modification. (**A**) A549 cells were transfected with siDCAF13#1, #2, #3 for 48 h. Before harvest, cells were treated with MG132 (5 μM) for 6 h, cell lysates were subjected to western blotting with indicated antibodies. (**B**) A549 cells were transfected with siDCAF13#1 for 48 h., Before harvest, cells were treated with 10 mg/mL cycloheximide (CHX) for the indicated time points, and then the cell lysates were used for western blotting. (**C**) The ubiquitinase and substrate website predicts that p53 is the most potentially substrate specifically recognized by DCAF13 using UbiBrowser database. (**D**) Immunofluorescence assay was performed to observe the cellular localization of DCAF13 and p53 in A549 and SPC-A1 cells. Scale bar = 50 μm. (**E**) A549 or SPC-A1 whole cell lysates were immunoprecipitated with protein-A/G agarose beads (mock IgG) or anti-p53 antibody and western blotted with indicated antibodies. Levels of endogenous p53, DCAF13, CUL4A, DDB1 and RBX1 were determined by western blot analysis of cell extracts (input) with indicated antibodies. **F-H**. A549 or SPC-A1 cells were co-transfected with the indicated siRNA and plasmids for 48 h. Before harvest, cells were treated with MG132 (5 μM) for 6 h. Subsequently, cell lysates were subjected to IP assays with p53 antibody for 12 h and then with protein G beads for 4 h. The mixtures including were ubiquitinated p53 were analyzed by western blot analysis with indicated antibodies. Polyubiquitination level of p53 was detected with the anti-His antibody. I. A549 or SPC-A1 cells were co-transfected with the indicated siRNA and plasmids, including K0 (lysineless), K48 (only K48-linked Ub), and K63 (only K63-linked Ub) as indicated, the following procedure is as shown in Fig. 6F-H

DCAF13 knockdown was found to be associated with an increase in p53 protein levels but not mRNA expression (Fig. 6A). Therefore, we hypothesized that DCAF13 might be involved in the degradation and stabilization of p53 protein. To verify our speculation, we treated A549 cells with the proteasome inhibitor MG132 and found that it antagonized the increase in p53 protein caused by DCAF13 knockdown. Additionally, we treated A549 cells with the protein synthesis inhibitor cycloheximide (CHX) and analyzed p53 stability upon DCAF13 knockdown. The results showed that the p53 protein half-life was significantly longer in DCAF13-knockdown A549 cells (Fig. 6B), indicating that DCAF13 regulates the protein stability of p53. Given that DCAF13 is a member of the CRL4 E3 ubiquitin ligase, we hypothesize that it might be involved in the ubiquitination modification of p53. Using the UbiBrowser database [47] (a comprehensive resource for proteome-wide known and predicted ubiquitin ligase/deubiquitinase–substrate interactions in eukaryotic species), we found that p53 was the most likely ubiquitinated substrate specifically recognized by DCAF13, with the highest confidence (Fig. 6C). To investigate whether DCAF13 modulates p53 through direct interactions, we performed immunofluorescence assays and demonstrated the intracellular co-localization of DCAF13 and p53 primarily in the nucleus in A549 or SPC-A1 cells (Fig. 6D). We also performed Co-IP assays and confirmed that p53 physically interacts with DCAF13 in A549 or SPC-A1 cells, with CUL4A, DDB1, and RBX1 being involved (Fig. 6E). Furthermore, we demonstrated that DCAF13 overexpression significantly upregulated p53 polyubiquitination levels in A549 and SPC-A1 cells (Fig. 6F), while DCAF13 knockdown downregulated p53 polyubiquitination levels in A549 and SPC-A1 cells (Fig. 6G-H). We also analyzed the specific type of ubiquitin chain generated on p53 by DCAF13. K48-linked ubiquitin chains are known to label their protein substrate for proteasomal degradation [48]. Using K0 (lysineless), K48 (only K48-linked Ub), and K63 (only K63-linked Ub) three plasmids, our results further suggest that knockdown of DCAF13 primarily downregulated K48-linked p53 protein ubiquitination and secondarily downregulated K63-linked p53 protein ubiquitination (Fig. 6I). Overall, these findings demonstrate that DCAF13 combines with p53 and promotes p53 protein degradation by upregulating p53 K48-linked polyubiquitination levels.

### Knockdown of DCAF13 inhibits LUAD cell growth in vivo

**Fig. 7:**
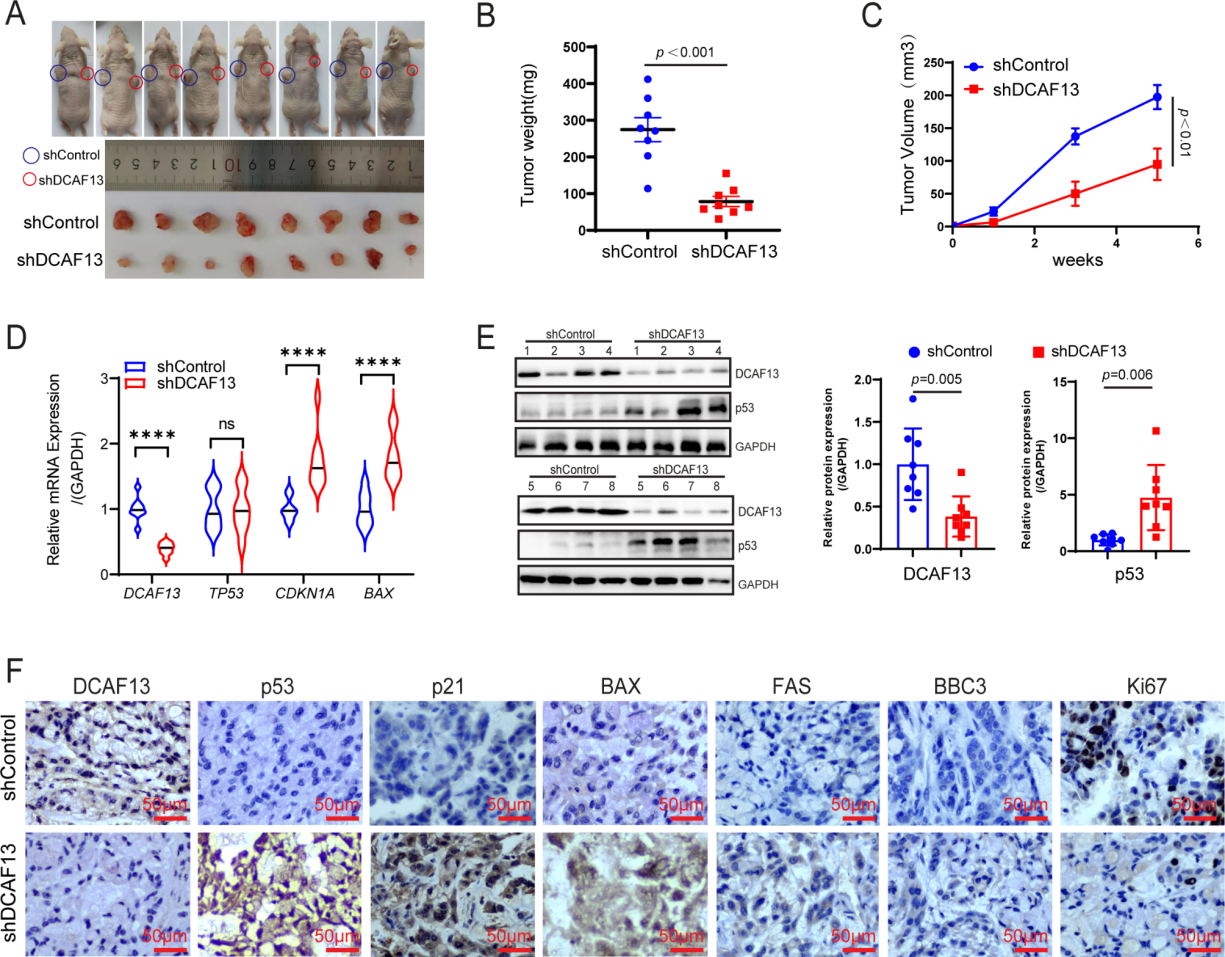
DCAF13 knockdown inhibits the LUAD cell growth in vivo. (**A**) A549 shDCAF13 or shControl cells were implanted into BALB/C nude mice by subcutaneous injection. Photograph was shown as the xenograft tumor with shControl or shDCAF13 A549 cells. Tumors were separated after 5 weeks. (**B**) Mean tumor weights in mice. (**C**) Xenograft tumor growth curves in mice. (**D**) The mRNA expression of *DCAF13, TP53, CDKN1A*, and *BAX* in mouse tissues were detected by qPCR. (**E**) The protein expression of DCAF13 and p53 in mouse tissues were detected by western blot analysis. Data were statistically analyzed using Student’s t-test and values are shown as mean ± SD. **** *p* < 0.0001. (**F**) The protein expression of DCAF13, p53, p21, BAX, FAS, BBC3 and Ki67 in mouse tumor tissues were detected by immunohistochemistry assay. Representative images are shown, scale bar = 50 μm

The results presented above suggest that DCAF13 plays a negative regulatory role in the p53 signaling pathway by modulating ubiquitination, thereby promoting the malignant biological behavior of LUAD cells. To further investigate the function of DCAF13 in vivo, we generated A549 cell lines infected with either shDCAF13 or shControl lentivirus and implanted them subcutaneously into BALB/c nude mice. Tumor formation assays in mice revealed that tumor size and weight were significantly reduced in the DCAF13 knockdown group compared to the control group (Fig. 7A-B). Additionally, the tumors in the DCAF13 knockdown group grew at a significantly slower rate than those in the control group (Fig. 7C). We also extracted mRNA and protein from the tumors to analyze the impact of DCAF13 knockdown on the p53 signaling pathway. The results showed that DCAF13 knockdown significantly increased the mRNA expression of *CDKN1A* and *BAX*, without affecting *TP53* mRNA expression (Fig. 7D). However, DCAF13 knockdown significantly increased p53 protein expression (Fig. 7E). Immunohistochemical staining further revealed that DCAF13 knockdown increased the protein expression of p53, p21, BAX, FAS, BBC3, and Ki67 (Fig. 7F). These results demonstrated that DCAF13 inhibited tumor growth associated with p53 protein degradation and signaling pathways.

### Inhibition of p53 could attenuate the effect of DCAF13 in A549 and SPC-A1 cells

**Fig. 8:**
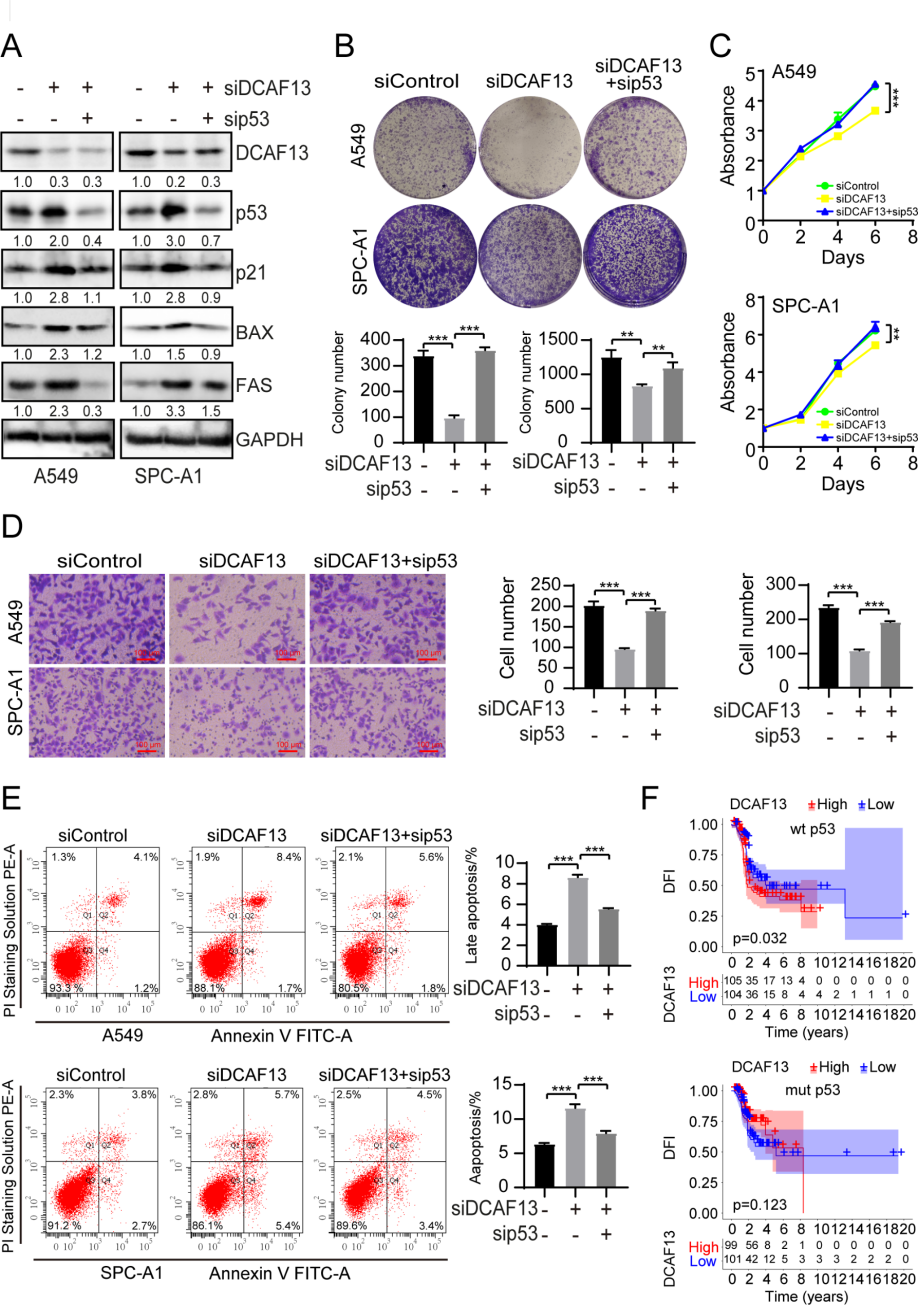
Inhibition of p53 could attenuate the effect of DCAF13 in A549 and SPC-A1 cells. (**A**) Protein expression of p53, p21, BAX, FAS were analyzed by Western blotting in A549 or SPC-A1 cells transfected with the indicated siRNAs. GAPDH was used as a control. (**B**) Cell clone formation experiments were performed in A549 and SPC-A1 cells transfected with the indicated siRNAs. (**C**) CCK-8 experiments were performed in A549 and SPC-A1 cells transfected with the indicated siRNAs. (**D**) Cell migration experiments were performed in A549 and SPC-A1 cells transfected with the indicated siRNAs. (**E**) Flow cytometry was used to analyze the apoptosis rates in A549 and SPC-A1 cells transfected with the indicated siRNAs. (**F**) Association between DCAF13 mRNA expression and prognosis in p53 wild-type or mutant type TCGA LUAD samples by Kaplan-Meier analysis. Student’s t-test was used in Fig. 8B-E. Data presented as mean ± SD, n = 3, * *p* < 0.05, ** *p* < 0.01, and *** *p* < 0.001. Scale bar = 100 μm

**Fig. 9:**
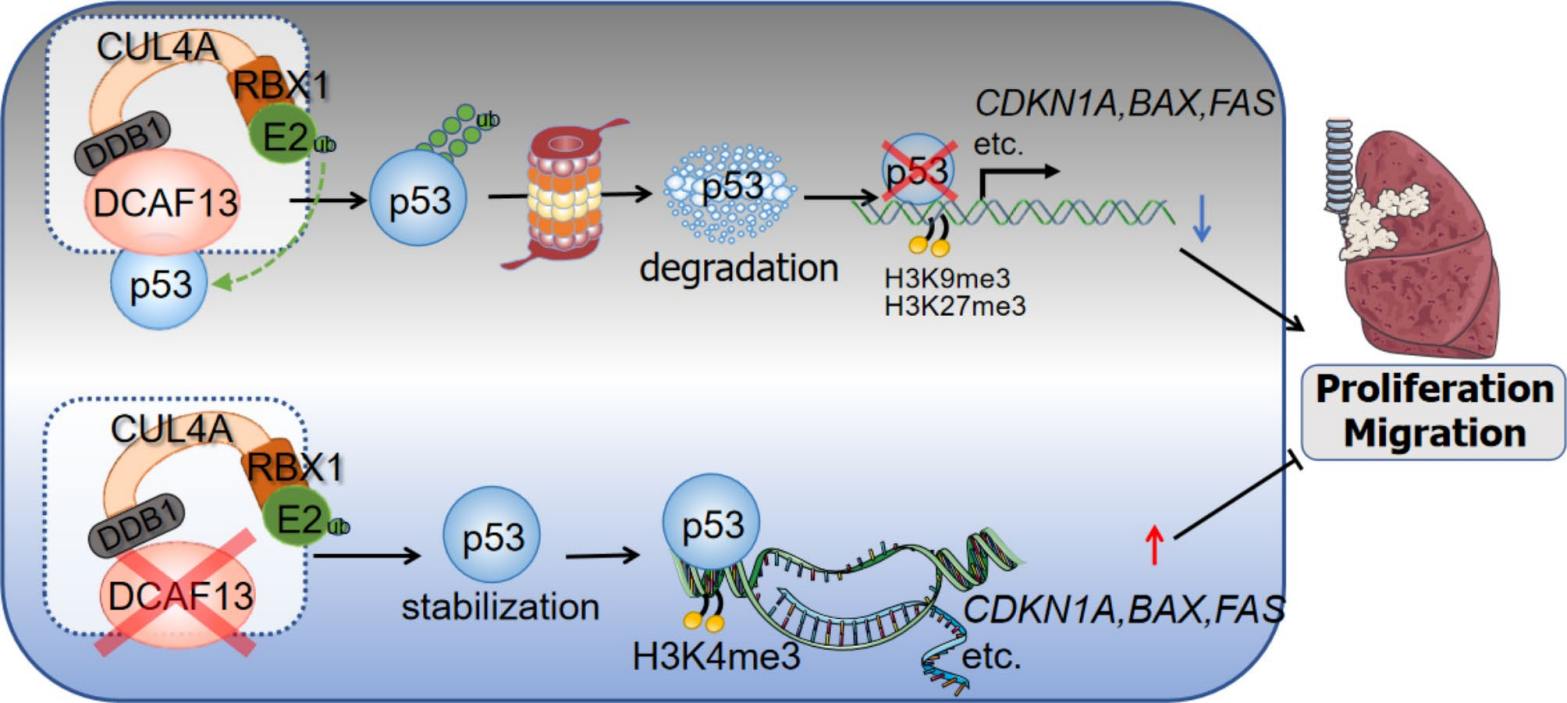
A model diagram of DCAF13 promotes p53 protein ubiquitination degradation via CRL4 complex, which in turn inhibits the p53 signaling pathway, leading to the altered histone modifications of *CDKN1A, BAX* and *FAS*, and ultimately promotes LUAD progression

In order to clarify the role of p53 in DCAF13 promoting lung adenocarcinoma, siRNA interference technology was used to knock down p53 and DCAF13 (Fig. 8A). As a result, the promotive effect of siDCAF13 on p53 downstream proteins was reduced upon p53 knockdown (Fig. 8A). In addition, we further investigated whether the effect of DCAF13 on lung adenocarcinoma cell function was dependent on p53. The results revealed that knockdown of p53 rescued the inhibitory effect of siDCAF13 on cell clone formation and cell growth (Fig. 8B-C). Similarly, the inhibitory effect of siDCAF13 on cell migration was also rescued by the knockdown of p53 (Fig. 8D). Moreover, knockdown of p53 attenuated the promotion of cell apoptosis by siDCAF13 (Fig. 8E). Finally, we proceeded to analyze the significance of DCAF13 as a prognostic marker in lung adenocarcinoma patients with either p53 wild-type or mutant type. The results indicated that DCAF13 was a poor prognostic marker for Disease Free Interval in p53 wild-type patients but not in p53 mutant patients (Fig. 8F). In general, we summarize a model to illustrate that DCAF13, as a substrate recognition protein in the CRL4 complex, incorporates with p53 and increases p53 polyubiquitination to repress the p53 signaling pathway, as well as inhibit downstream protein p21 and BAX etc. expression. DCAF13 knockdown promotes p53 protein stabilization and inhibits the proliferation and migration of lung adenocarcinoma cells (Fig. 9).

## Discussion

Lung cancer is the most lethal tumor worldwide, and p53, as the most famous tumor suppressor participates in the pathogenesis and development in lung cancer. Thus, an adequate disclosure of the p53 signaling pathway is anticipated to deliver potential therapeutic objectives for lung cancer. In this study, DCAF13 has been identified to be an oncogenic factor that can promote LUAD progression. Firstly, DCAF13 expression is elevated in LUAD tissues and negatively associated with the clinical prognosis in LUAD patients. Secondly, we provide evidence that DCAF13 acts as a substrate receptor in CRL4 E3 ligase and binds directly onto p53 as its ubiquitinated substrate. Mechanistically, DCAF13 knockdown inhibits p53 ubiquitinated degradation, and promotes the p53 signaling pathway, including p53 downstream target proteins p21, BAX, FAS, BBC3, PERP, etc. Moreover, DCAF13 depletion inhibits the malignant biological behavior of LUAD cells, which provides a theoretical basis for the design of DCAF13-based drugs in anti-pulmonary tumor therapy.

DCAFs are members of the CRL4 family of E3 ubiquitin ligases, which can conjugate distinct substrates so as to exert various functions, such as tumor progression, DNA repair, cell cycle progression, adipogenesis, cell division, and differentiation [49]. In common, CRL4-DCAF1 E3 ligase degrades histone deacetylase SIRT7 [50], and then CRL4-DCAF2 specifically recognizes proliferating cell nuclear antigen (PCNA), thereby permitting the cell cycle and DNA repair procedures to be accomplished sequentially [51]. In addition, CRL4-DCAF3 targets both CDK2 and CDK4/6 cell cycle proteins for degradation [52]. CRL4-DCAF6 ubiquitinates and degrades CtBP1/2, causing BBC3-dependent apoptosis [53], and CRL4-DCAF8 complex targets the chromatin remodeler LSH and coordinates the epigenetic inheritance of ferroptosis genes [54]. CRL4-DCAF12 binds and ubiquitinates monomeric CCT5, a subunit of the TRiC chaperonin [55]. Here, we discovered that DCAF13 interacts with p53 in LUAD cells. The ubiquitinase-substrate prediction website also suggests that p53 may be a substrate of DCAF13. Our further experiments confirmed that DCAF13 knockdown inhibits p53 protein ubiquitination via the proteasome, demonstrating that the DCAF13-CRL4 complex targets p53 ubiquitination degradation to serve as a significant role in the development of LUAD.

Previous studies have shown that DCAF13 is involved in promoting tumor growth via the induction of protein or mRNA degradation [22, 23]. Consistent with these reports, our previous work also tentatively suggests that DCAF13 is a potential oncogene in LUAD [56]. Significantly, our study has further analyzed this critical role and revealed that DCAF13 promotes LUAD cell growth and migration by ubiquitinating and degrading p53. A recent study by Shan demonstrates that DCAF13 promotes breast cancer cell proliferation by facilitating polyubiquitination of PERP, which acts as the transcriptional downstream protein of p53 [24]. Consistent with Shan ‘s study, we found that knockdown of DCAF13 resulted in mRNA accumulation of *PERP*. In addition, we elucidate a new molecular mechanism by which DCAF13 targets polyubiquitination and degradation of p53. Together, these findings broaden our understanding of the functions and mechanisms of DCAF13 in tumors and provide a stronger theoretical basis for considering DCAF13 as a potential target for tumor therapy.

The p53 tumor suppressor protein is dysfunctional in most malignancies, including lung cancer, leading to the inactivation of its anti-tumor properties [57, 58]. The stability of p53 protein is critical in tumors, and its regulatory network is complex, with multiple genes involved in p53 ubiquitination degradation and stabilization. One important mechanism is the mouse double minute 2 (MDM2)/mouse double minute 4 (MDM4)-mediated degradation of wild-type p53 [59, 60]. In addition, the E6/E6-associated protein (E6AP) complex and tripartite motif-containing 28 (TRIM28) also target p53 ubiquitination degradation [61, 62], while the protein interacting with NIMA-1 (PIN1) binds to p53, preventing p53 degradation by MDM2 [63]. We have identified that DCAF13 also serves as a moderator to target p53 ubiquitination degradation, which involves in CUL4A and RBX1 of the CRL4 complex. Additionally, DCAF13 knockdown mainly downregulates the K48-linked ubiquitin type of p53. Theoretically, our discoveries broaden the knowledge about the post-translational modification mechanism of p53.

Besides, DCAF13 inhibition stabilizes p53 protein and promotes a series of tumor suppressor genes. Furthermore, small molecule inhibitors that inhibit MDM2-p53 binding, such as Nutlin derivatives, APG-115, and AMG 232, have entered clinical trials as a tool to stabilize p53 and restore its potency (e.g., NCT02935907, NCT03611868, NCT04785196, NCT03781986, NCT03634228, NCT04116541, NCT04022876, NCT03725436, NCT03654716). In combination with these findings, we can propose that DCAF13 inhibition can be used to hinder the growth of LUAD cells in vivo by downregulating p53 protein stabilization and signaling pathway. Clinically, targeting DCAF13 may be a novel therapeutic strategy for p53 protein stability in LUAD. The question of whether there is a synergistic or antagonistic effect between DCAF13 and MDM2 in regulating p53 degradation will be the subject of future studies.

Histone modifications play a crucial role in the regulation of gene transcriptional activation and deactivation [64]. Our study revealed that DCAF13 knockdown resulted in the inhibition of H3K9me3 and the promotion of H3K4me3 on p53-RE in the promoter regions of *BAX, CDKN1A, FAS*, and *PIDD1*, as well as the inhibition of H3K27me3 on p53-RE in the promoter regions of *BAX* and *CDKN1A*. Currently, the regulation of histone modifying enzymes by DCAF13 is limited to early embryonic development [16]. DCAF13-CRL4 was reported to target suppressor of variegation 3–9 homolog 1 (SUV39H1) for polyubiquitination and proteasomal degradation, which facilitated H3K9me3 removal and zygotic gene expression [16]. According to our experimental results, we speculate that other histone modifying enzymes may participate in the regulation of *BAX, CDKN1A, FAS*, and *PIDD1* expression by DCAF13. However, further investigation is required to determine which histone modification enzymes are responsible for the altered histone modification of the promoter regions in these genes by DCAF13.

There are also some limitations in our study. For instance, the molecular mechanism of elevated DCAF13 expression in LUAD is not yet understood. Additionally, we did not validate all p53 downstream target genes regulated by DCAF13. Furthermore, since we have previously found that DCAF13 knockdown inhibits cell growth and migration in the p53-deficient cell line NCI-H1299 [56], here we found that DCAF13 inhibits *CYCS* expression in NCI-H1299 cells, suggesting that p53 may not be the only active substrate of DCAF13 in LUAD, this may be the reason why DCAF13 also promotes cell proliferation and migration in NCI-H1299 cells. More mysterious substrates of DCAF13 remain to be discovered and thus to be identified.

## Conclusion

Generally, protein ubiquitination degradation plays a critical role in LUAD progression. Theoretically, our study demonstrates that high DCAF13 expression is a prognostic risk factor for LUAD. Mechanically, DCAF13-CRL4 targets p53 polyubiquitination and proteasomal degradation to inhibit p53 protein stabilization, negatively regulating the tumor-suppressive p53 signaling pathway, altering gene histone modifications, and ultimately promote LUAD cell growth and migration in LUAD. In summary, we identified a previously unknown role of DCAF13 in promoting LUAD, providing an experimental basis for prognosis determination and development of targeted therapies in LUAD. Our findings help to further develop novel pharmacological strategies in the future.

### Electronic supplementary material

Below is the link to the electronic supplementary material.

**Supplementary Material 1: Supplementary Table S1** Materials

**Supplementary Material 2: Supplementary Table S2** Differential genes with p-adjust < 0.05 and fold change > 2

**Supplementary Material 3: Supplementary Figure S1** High expression of DCAF13 is not associated with TP53 mutation. **Supplementary Figure S2** The overexpression of DCAF13 promotes malignant progression of lung adenocarcinoma cells. **Supplementary Figure S3** Relative mRNA expression of *CASP3*

## Data Availability

Data generated in this study are available from the corresponding author upon reasonable request.

## Abbreviations

CRBN: Cereblon
CRL4: Cullin 4-RING ligase
CUL4A: Cullin 4 A
CUL4B: Cullin 4B
DCAF13: DDB1 and CUL4 associated factor 13
DDB1: Damage specific DNA binding protein 1
DFI: Disease Free Interval
E6AP: E6-associated protein
GTEx: Genotype-Tissue Expression
H3K27me3: Tri-methylation of histone H3 at lysine 27
H3K4me3: Tri-methylation of histone H3 at lysine 4
H3K9me3: Tri-methylation of histone H3 at lysine 9
IHC: Immunohistochemistry
LUAD: Lung adenocarcinoma
MDM2: Mouse double minute 2
NSCLC: Non-small cell lung cancer
p53-RE: P53 transcription factor binding response elements
PI3K: Phosphatidylinositol 3-kinase
PIN1: Protein interacting with NIMA-1
PTEN: Phosphatase and tensin homolog
RBX1: RING-box protein 1
SUV39H1: SUV39H1 histone lysine methyltransferase
TCGA: The Cancer Genome Atlas
TRIM28: Tripartite motif-containing 28
UALCAN: The University of ALabama at Birmingham CANcer data analysis Portal
