# Supplementary material for: DCAF13 inhibits the p53 signaling pathway by promoting p53 ubiquitination modification in lung adenocarcinoma
Source: J Exp Clin Cancer Res. 2024 Jan 2;43:3. doi: 10.1186/s13046-023-02936-2 (PMC10759521; doi:10.1186/s13046-023-02936-2)
Supplement: Supplementary file 3 — Supplementary Material 3: Supplementary Figure S1 High expression of DCAF13 is not associated with TP53 mutation. Supplementary Figure S2 The overexpression of DCAF13 promotes malignant progression of lung adenocarcinoma cells. Supplementary Figure S3 Relative mRNA expression of CASP3 [file 13046_2023_2936_MOESM3_ESM.docx]

Supplementary Figures

Supplementary Figure S1 DCAF13 was significantly overexpressed in TP53-mutated or non-mutated lung adenocarcinoma tissues compared to normal lung tissues.


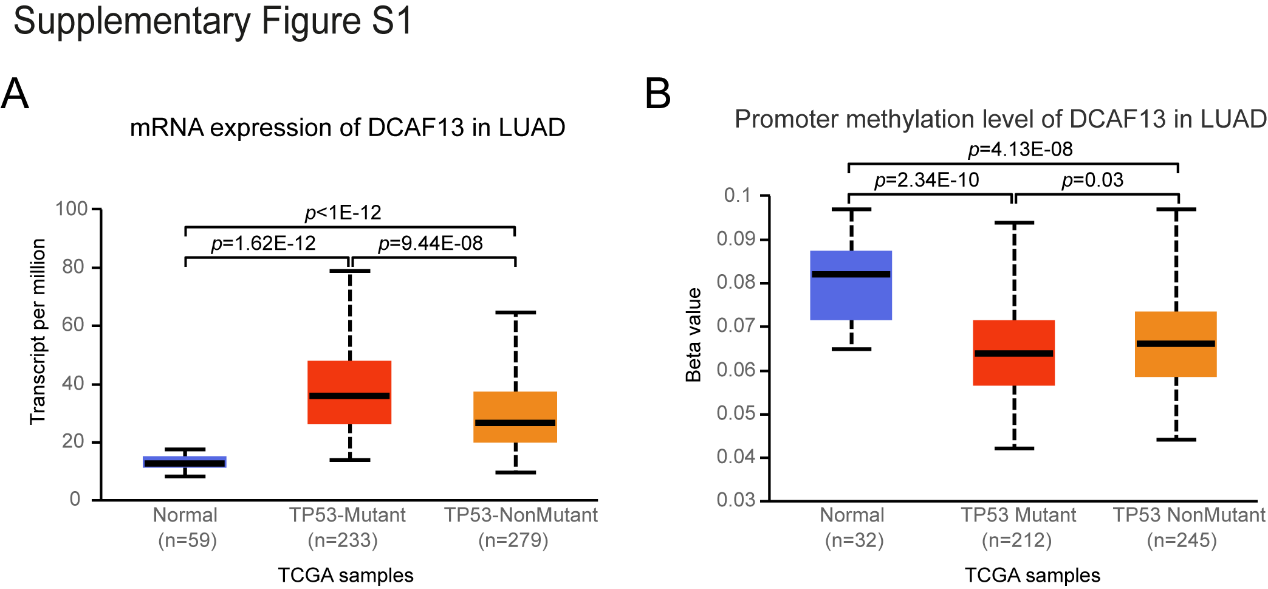
 Supplementary Figure S1. The mRNA expression and promoter methylation level of DCAF13 were analyzed by UALCAN. Differences in significance were marked (Mann-Whitney U test).

Supplementary Figure S2 The overexpression of DCAF13 promotes malignant progression of lung adenocarcinoma cells.


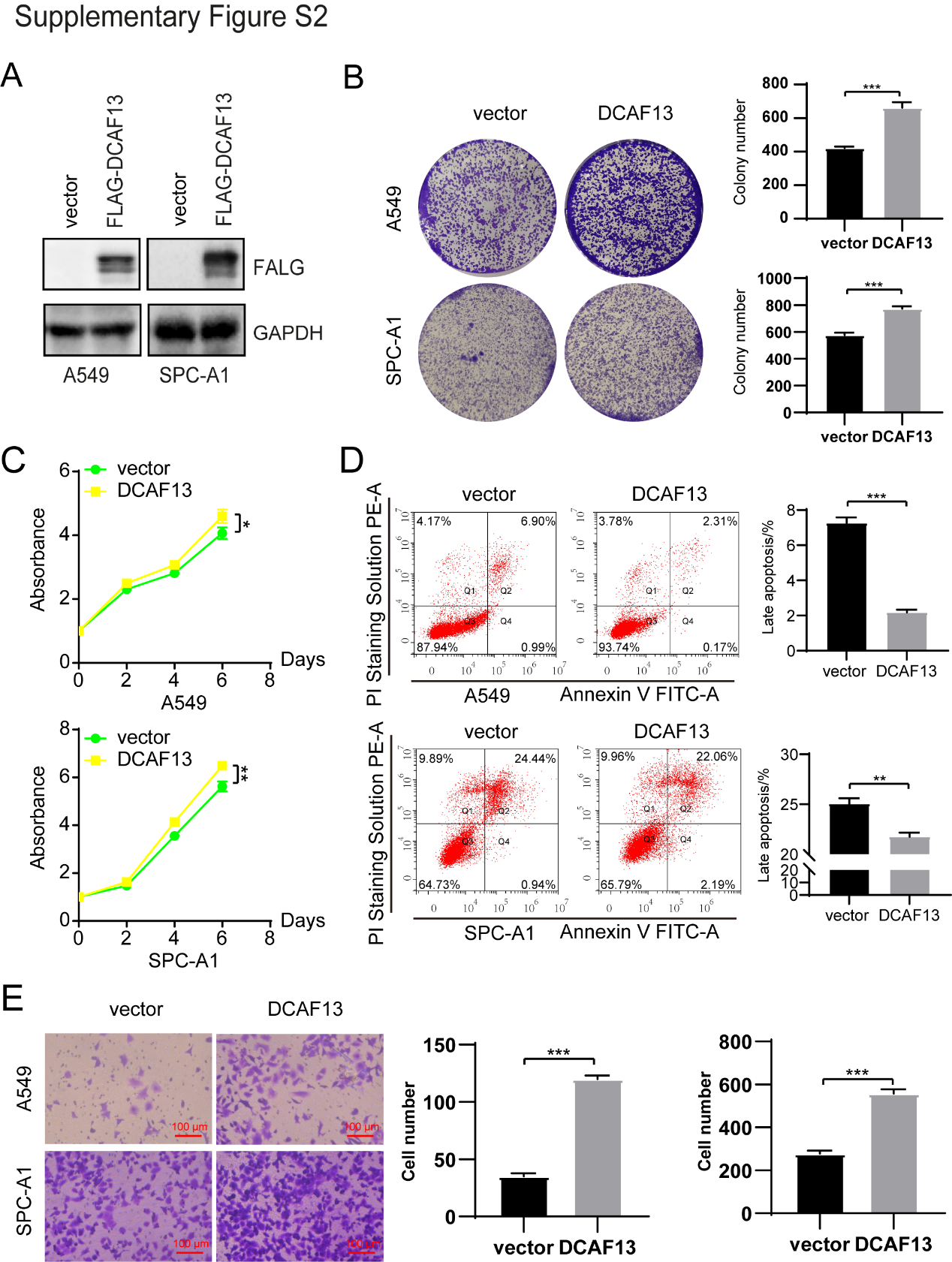


Supplementary Figure S2. The overexpression of DCAF13 promotes LUAD cell growth, migration and apoptosis inhibition in *vitro*. A. DCAF13 overexpression was detected by western blotting in A549 and SPC-A1 cells. B. Cell clone formation assays were performed following DCAF13 overexpression in A549 and SPC-A1 cells. C. CCK-8 assays were performed following DCAF13 overexpression in A549 and SPC-A1 cells. D. Flow cytometry was used to analyze the apoptosis rates following DCAF13 overexpression in A549 and SPC-A1 cells. E. Cell migration assays were performed following DCAF13 overexpression in A549 and SPC-A1 cells. Student's t-test was used in Supplementary Figure S2B-E. Data presented as mean ± SD, n = 3, * *p* <0.05, ** *p* <0.01, and *** *p* <0.001. Scale bar = 100 μm.

Supplementary Figure S3 Relative mRNA expression of *CASP3*


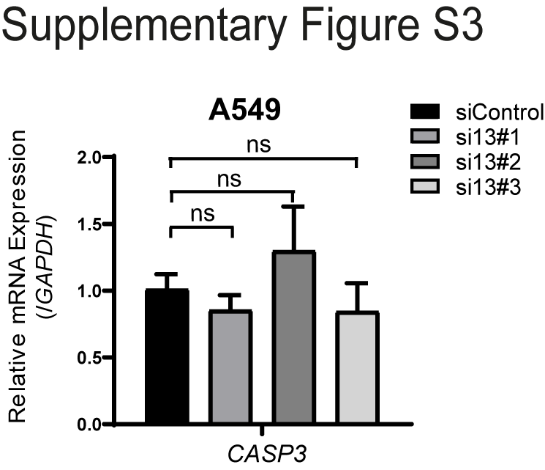


Supplementary Figure S3. The mRNA expression of *CASP3* was detected by RT-qPCR in A549 cells transfected with siControl or the indicated siRNAs.
